# Intrathecal chemotherapy for leptomeningeal metastasis of non-small-cell lung cancer: a systematic review

**DOI:** 10.3389/fphar.2026.1788293

**Published:** 2026-06-25

**Authors:** Dan Wang, Wei Liu, Lingli Zheng, Yan Li, Ting Jiang, Jing Li

**Affiliations:** 1 Clinical Medical College, Chengdu Medical College, Chengdu City, Sichuan Province, China; 2 Department of Pharmacy, The First Affliated Hospital of Chengdu Medical College, Chengdu City, Sichuan Province, China; 3 Department of Oncology, The First Affliated Hospital of Chengdu Medical College, Chengdu, China; 4 Department of Oncology, The First Affiliated Hospital of Traditional Chinese Medical of Chengdu Medical College-Xindu Hospital of Traditional Chinese Medical, Chengdu, Sichuan, China

**Keywords:** adverse events, intrathecal injection, leptomeningeal metastasis, non-small-cell lung cancer, systematic review

## Abstract

**Background:**

Leptomeningeal metastasis (LM) is a severe complication of advanced non-small-cell lung cancer (NSCLC) that is increasingly detected due to improved diagnostics. Intrathecal therapy, which involves delivering drugs directly into the subarachnoid space, has become a key treatment approach. A variety of cytotoxic drugs, targeted therapies, immune-checkpoint inhibitors, and combination treatments are now available through different intrathecal protocols, offering new hope to patients.

**Methods:**

Extensive searches were conducted in PubMed, Embase, the Cochrane Library, Web of Science, Wanfang Data, CNKI, and VIP. Data collected included histological subtype, intrathecal delivery method, treatment regimen, median progression-free survival (mPFS), median overall survival (mOS), objective response rate (ORR), disease control rate (DCR), and adverse events (AEs) in ≥10% of patients.

**Results:**

Twelve clinical studies with 544 patients (average age 53.27 years) met the criteria. Drug delivery was via lumbar puncture or Ommaya reservoir. Objective response rates were 34.5%–86.4%, and disease control rates were 62.5%–94.9%. Three trials reported progression-free survival of 3.5, 6.3, and 9.6 months, with overall survival from 3.66 to 17 months. AEs in over 10% of patients included myelosuppression, gastrointestinal issues, and neurological symptoms.

**Conclusion:**

Intrathecal therapy offers significant clinical benefits, especially when combined with other treatments, with a manageable toxicity profile.

## 1 Introduction

LM also known as leptomeningeal carcinomatosis or neoplastic meningitis, is a serious complication affecting 3%–9.4% of advanced NSCLC patients, with autopsy reports suggesting a prevalence of up to 25%. Patients with specific driver mutations are at higher risk. Improved diagnostic methods like Cerebrospinal Fluid (CSF)cytology and liquid biopsy allow for earlier detection, but LM still has a poor prognosis due to the adaptability of cancer cells in the challenging CSF environment (Gion et al., 2017; Lung Cancer Medical Education Committee of Chinese Medical Education Association, 2026). Without treatment, NSCLC patients with LM have a median survival of 2–4 months. Conventional treatments extend this to 4–6 months, while targeted therapies can improve survival further. Death typically results from neurological decline rather than systemic cancer progression (Kang Xun, 2024; Zhao et al., 2023). The average time from initial NSCLC diagnosis to leptomeningeal seeding is 8–15 months, but targeted and immune therapies have extended this period, with cases now often appearing after 3 years. Risk factors include patient age, primary tumor size, other metastatic sites (liver, bone, pleura, adrenal), and biomarkers like EGFR mutation, high calcium, and carcinoembryonic antigen (He et al., 2021; Li et al., 2022; Zheng et al., 2026). Despite various treatments, LM in NSCLC remains a challenge, with only slight improvements in survival and quality of life. The blood-brain barrier limits CSF drug exposure to less than 5% of plasma levels, protecting the central nervous system (D’Aiello et al., 2023; Zhao et al., 2023; Wang et al., 2021; Brastianos et al., 2020). Intrathecal chemotherapy (ITC) bypasses this barrier by delivering drugs directly into the subarachnoid space, achieving higher CSF concentrations, extending survival, and alleviating neurological symptoms (Li et al., 2020). Previous studies have shown that ITC therapy significantly extends median survival and improves symptoms compared to whole-brain radiotherapy, EGFR-TKIs, systemic chemotherapy, or immune-checkpoint blockade (Wu et al., 2016; Mills et al., 2024). However, due to limited prospective clinical data, mainly from retrospective series and case reports, a standardized intrathecal regimen or treatment duration for NSCLC with LM has not been established. Consequently, ITC is typically reserved for patients unresponsive to systemic therapies (Liang Xh and Zhan, 2019). This study aims to provide an evidence-based treatment guideline for NSCLC patients with LM eligible for intrathecal treatment.

## 2 Methodology

### 2.1 Study selection

The databases of scientific literature, including PubMed, Embase, Cochrane library, Web of Science, China Wanfang, China CNKI, China VIP and China Wan fang were searched for relevant articles published as of 5 January 2026. The free words and subject headings search method was used. The following keywords were used for the search: “non-small-cell lung cancer” or “Non-Small-Cell Lung Carcinomas” and “leptomeningeal metastases” and “Intrathecal Injection” or “intrathecal therapy”.

### 2.2 Inclusion criteria and exclusion criteria

**Inclusion criteria**: (1) A pathological assessment confirming a NSCLC. (2) leptomeningeal metastases. (3) In every outcome, at least one of the following metrics was available or could be derived: ORR, DCR, PFS, OS, and SAE occurrences. **Exclusion criteria**: Basic research, conference summaries, repeated publications of the same material by the same center and author, and non-original articles like meta-analyses and reviews, and analyses in which crucial data cannot be gathered.

### 2.3 Data extraction

The search strategy was collaboratively established by all authors. Two researchers independently executed the literature search, cross-referenced the retrieved literature, selected appropriate trials, and extracted data. In instances of disagreement, discussions were held among the team until consensus was reached. The collected data encompassed the first author, publication year, country, clinical trial design description, sample size, evaluation criteria for efficacy and adverse events, ORR, DCR, PFS, OS, and AEs.

### 2.4 Quality assessment

An independent evaluation of the literature’s quality was conducted by two researchers, with studies assessed according to the Methodological Index for Non-Randomised Studies (MINORS). Discrepancies in quality assessment were resolved through team discussions, leading to a consensus.

## 3 Results

### 3.1 Literature search and quality appraisal

**FIGURE 1:**
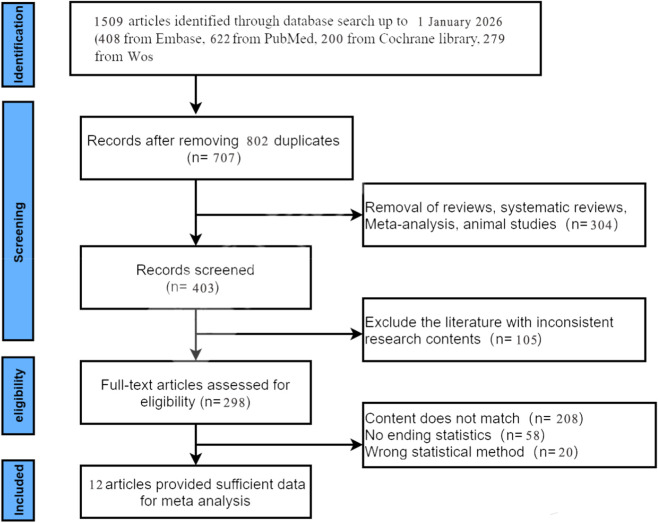
Literature screening process.

Following a systematic search that initially identified 1,509 citations, three successive screening rounds refined the evidence base to twelve clinical studies. These comprised five single-arm phase I trials, three single-arm phase II trials, and seven retrospective cohort studies, collectively enrolling a total of 544 patients. All participants were diagnosed with LM secondary to NSCLC, confirmed through histopathological examination and CSF cytology (Figure 1). Detailed data were extracted on variables such as mean age, treatment protocols, efficacy endpoints, and AEs profiles. Eight studies reported the ORR, seven provided the DCR, three included PFS, and eleven detailed OS. A comprehensive quality appraisal and the extracted variables are systematically presented in Table 1.

**TABLE 1:** General information of the included literature.

Author	Year	Study type	Nation	Number	Average age	Therapeutic regimens	ORR	DCR	PFS	OS	AE (≥10%)	MINORS score
Fan CJ	2021	PhaseI/II,single-arm	China	30	54 (31–70)	Intrathecal pemetrexed 50 mg	84.60%	NA	NA	9.0(6.6–11.4)	Myelosuppression (36.7%),emesis (20.0%), limb pain (16.7%), headache (10.0%)	14
Oliva IG	2022	PhaseI,single-arm	United States	25	43 (30–73)	Intrathecal nivolumab 50 mg plus intravenous nivolumab 240 mg every 14 days; select patients received concomitant BRAF/MEK inhibitor therapy	NA	NA	NA	4.9 (3.2–6.6)	Gastro-intestinal events (36%), transaminase elevation (20%), headache (20%), pruritus (16%), arthralgia (16%)	13
Li HY	2022	PhaseI,single-arm	China	23	54 (36–68)	Pemetrexed 30 mg, 40 mg, or 50 mg was administered via ommaya reservoir on days 1 and 8 of a 21-day cycle	43.5% (23.2–63.8)	82.6% (61.2–95.0)	6.3 (0.8-NA)	9.5 (2.9- Na)	Myelosuppression (34.8%), transaminitis (26.1%), anaemia (17.4%)	14
Hou L	2021	PhaseI,single-arm	China	29	55 (23–70)	Methotrexate 10 mg reconstituted in 10 mL buffered diluent: Induction twice weekly for 4 weeks, consolidation weekly for 4 weeks, maintenance monthly until death	34.5% (17.9–54.9)	68.97% (50.1–84.0)	NA	6.32 (4.3–8.33	Myelosuppression (10.34%)	12
Hou L	2021	PhaseI,single-arm	China	24	55 (23–70)	Methotrexate 15 mg: induction twice weekly for 4 weeks, consolidation once weekly for 4 weeks, and maintenance once monthly until death	62.5% (40.6–81.2)	62.5% (40.6–81.2)	NA	3.66 (2.28–5.04)	Myelosuppression (10.34%)	12
Fan CJ	2025	PhaseI,single-arm	China	19	53 (39–73)	Intrathecal sintilimab 40 mg plus intrathecal dexamethasone 1 mg every 21 days for six cycles, followed by maintenance every 28 days until progression or intolerable toxicity	38.9% (17.3–64.3)	72.2% (46.5–89.8)	3.5 (2.7–4.2)	11.5 (0.0–25.4)	Rash (21.1%), gastrointestinal toxicity (15.8%), limb paresthesia (10.5%), hyperthyroidism (10.5%), pruritus (10.5%), cervical pain (10.5%)	13
Fan C	2024	PhaseII,single-arm	China	132	52 (31–74)	Intrathecal pemetrexed 50 mg: induction on days 1 and 5 of week 1, consolidation every 3 weeks for four cycles, then monthly maintenance until progression, intolerance, or adverse events; systemic therapy—predominantly third-generation EGFR-TKI—was continued unchanged for at least the first 21 days of intrathecal treatment	80.3% (72.9∼86.5)	94.7% (90.1∼97.5)	NA	12.0 (10.4–13.6)	Myelosuppression (31.8%)	15
Huang SJ	2024	Retrospective	China	21	54 (35–79)	Intrathecal methotrexate plus tyrosine-kinase inhibitor	NA	NA	NA	17 (6.5–27.5)	Myelosuppression (78.4%),gastrointestinal morbidity (40.5%)	14
Goyal G	2024	Retrospective	India	33	52.5 (21–75)	Monotherapy (n = 9), intrathecal therapy plus systemic treatment (n = 24) (targeted agents, chemotherapy, or their combination). Agents: methotrexate (n = 23), triple regimen (methotrexate + cytarabine + hydrocortisone) (n = 10)	NA	NA	NA	4.73 (2.33–8.16)	NA	12
Hong YP	2023	Retrospective	China	57	56 (49–62)	Intrathecal pemetrexed 10 mg, escalated to 20 mg or 30 mg (ceiling 50 mg) in non-responders, administered once to twice weekly until intracranial progression, unacceptable toxicity, or treatment discontinuation for any other cause	NA	NA	NA	13.2 (10.8–15.6)	Anemia (61.4%), thrombocytopenia (40.4%),leukopenia (33.3%)	13
Miao Q	2020	Retrospective	China	23	53 (38–74)	All patients received weekly intrathecal pemetrexed 10 mg plus dexamethasone 5 mg (2 mL) until two consecutive negative CSF cytologies, intolerable toxicity, or disease progression	34.8% (17.7–56.4)	82.6% (62.3–94.2)	9.6 (3.4–15.8)	NA	Myelosuppression (26.08%)	13
Pan ZY	2023	Phase II, single-arm	China	53	55 (28–73)	Induction: Intrathecal methotrexate 15 mg or cytarabine 50 mg twice weekly for up to four doses. Consolidation: Involved-field radiotherapy delivered concurrently with intrathecal chemotherapy	NA	NA	NA	6.5 (5.3–7.7)	Radiculitis (13%), mucositis (11%), leukoencephalopathy (23%)	14
Pan ZY	2016	Phase II, single-arm	China	59	55 (31–72)	Concurrent therapy comprised weekly intrathecal methotrexate 12.5–15 mg plus dexamethasone 5 mg coupled with involved-field irradiation	86.40%	94.90%	NA	6.5 (0.4–36.7)	Radiculitis (27%), myelosuppression (22%), mucositis (20%),leukoencephalopathy (68%),encephalopathy (19%)	14

Patients undergoing intrathecal therapy had a mean age of 53.27 ± 3.058 years, with an age range of 43–56 years. The treatment regimens included monotherapy, combination ITC, chemo-immunotherapy, intrathecal-plus-systemic strategies, and intrathecal chemoradiotherapy, utilizing agents such as pemetrexed, Methotrexate (MTX), cytarabine, nivolumab, and sintilimab. The routes of administration were lumbar puncture and Ommaya reservoir. Objective response rates varied from 34.5% to 86.4%; the lowest rate was observed with intrathecal MTX monotherapy, while rates of 80% or higher were achieved when intrathecal therapy was combined with oral targeted agents or irradiation. Disease-control rates ranged from 62.5% to 94.9%, with the highest rate achieved through intrathecal therapy combined with radiotherapy, followed—when feasible—by consolidative or maintenance ITC, systemic chemotherapy, or molecularly targeted treatment. Progression-free survival was infrequently reported, with durations of 3.5, 6.3, and 9.6 months. Overall survival ranged from 3.66 to 17 months, with the shortest duration observed with intrathecal MTX alone and the longest with the combination of intrathecal MTX and an oral tyrosine-kinase inhibitor. AEs observed in 10% or more of patients across various studies included myelosuppression, gastrointestinal toxicity, leukoencephalopathy, chronic encephalopathy, radiculitis, limb pain, headache, pruritus, and cutaneous reactions.

## 4 Discussion

### 4.1 Clinical manifestations and pathogenesis

The European Association of Neuro-Oncology and the European Society for Medical Oncology recommend intrathecal therapy for patients with LM who have an intact blood–cerebrospinal fluid barrier (Le Rhun et al., 2023). Similarly, the guidelines from the National Comprehensive Cancer Network support the use of intrathecal therapy in LM patients with a Karnofsky Performance Status of ≥60%, a modest tumor burden, non-disabling neurological deficits, and the capacity to tolerate systemic treatment (Horbinski and Portnow, 2024). In contrast to parenchymal metastases, leptomeningeal tumor cells exhibit both adhesive and free-floating growth patterns, presenting as either sessile nodules attached to the leptomeninges or as anchorage-independent clusters within the CSF (Lung Cancer Medical Education Committee of Chinese Medical Education Association, 2026). The exact pathogenesis of LM remains incompletely elucidated. However, it is postulated that neoplastic cells may infiltrate the subarachnoid space through various mechanisms, such as hematogenous dissemination, perivascular lymphatic migration, intra- or perineural invasion, or direct seeding from intracerebral or calvarial sources. Once within the subarachnoid space, these cells may disperse along the meninges through the flow of CSF (Zheng et al., 2026). Preclinical studies have demonstrated that tumor-secreted complement component 3 (C3) activates C3a receptors on the choroid plexus epithelium, compromising the integrity of the blood-brain barrier. This disruption permits the entry of plasma-derived amphiregulin and other mitogens into the CSF, thereby accelerating neoplastic growth. Pharmacological intervention targeting C3 signaling has consequently proven therapeutically efficacious in suppressing LM (Boire et al., 2017). The clinical manifestation of LM is marked by a diverse spectrum of symptoms, which are influenced by the neuroanatomical localization of malignant cells. These symptoms encompass asthenia, cephalalgia, vertigo, nausea and emesis, cranial nerve pareses presenting as diplopia or visual obscuration, sensorineural hearing loss, altered mental status, convulsive seizures, and focal neurological deficits. The molecular subtypes of NSCLC influence metastatic patterns, with approximately one-third of tumors possessing driver mutations showing a marked propensity for leptomeningeal dissemination. Patients with EGFR mutations exhibit a 9.4% incidence of LM, compared to 1.7% in those with wild-type EGFR. Upon the onset of leptomeningeal disease, the median overall survival for this molecular subgroup is 8.7 months (Nosaki et al., 2020). Research has identified several factors as independent determinants of the risk of LM, including age, tumour diameter, hepatic metastasis, osseous metastasis, pleural involvement, adrenal seeding, EGFR mutation status, serum calcium concentration, and carcinoembryonic antigen level as independent determinants of leptomeningeal-metastasis risk (He et al., 2021; Li et al., 2022).

### 4.2 Therapeutic selection

The 2025 Version 3 of the NCCN Guidelines for Central Nervous System Cancers authorizes the use of intrathecal thiotepa, MTX, cytarabine, topotecan, and etoposide. Additionally, it specifically recommends intrathecal pemetrexed for the treatment of EGFR-mutant NSCLC (Nabors and Baehring, 2025). ITC represents an active treatment modality for LM originating from NSCLC. However, the limited number of prospective trials—most evidence is derived from retrospective studies—leaves the optimal choice of agent, dosage, and treatment duration unresolved. Although MTX, cytarabine, and thiotepa are the most established and frequently used compounds, none have demonstrated clear superiority in any intrathecal regimen. Recently, there has been growing interest in the intrathecal administration of pemetrexed, targeted agents, cytokines, and immune checkpoint inhibitors for the treatment of NSCLC.

### 4.3 Intrathecal injection of chemotherapy drugs

MTX primarily exerts its pharmacological effects during the S phase of the cell cycle by inhibiting dihydrofolate reductase, with a CSF half-life ranging from approximately 4.5–8.0 h. Intrathecal administration of MTX results in a tenfold increase in plasma concentration 24 h post-administration compared to equivalent oral or intravenous doses. Moreover, the plasma absorption of intrathecally administered MTX is 20–30 times greater than that of the same dose administered intravenously for systemic therapy (Kang Xun, 2024; Wu Xi and Xiao, 2019). Although intrathecal methotrexate has been widely employed for the treatment of leptomeningeal disease in solid tumors, existing evidence in the context of lung cancer is derived primarily from small or retrospective cohorts. Consequently, optimal dosage, combination regimens, patient selection, and treatment cycles for NSCLC-LM remain to be established through large, prospective trials. Pemetrexed, a multitargeted antifolate, inhibits neoplastic cell proliferation by disrupting folate-dependent nucleotide biosynthesis. Emerging research is investigating its direct intrathecal application for the treatment of NSCLC leptomeningeal disease (Sun et al., 2010; Fan et al., 2021; Li et al., 2023; Zhou et al., 2023; Hong et al., 2023). Murine studies have demonstrated a biphasic elimination pattern for intrathecal pemetrexed, characterized by initial and terminal half-lives of 0.43 h and 1.43 h, respectively. These findings establish 1 mg/kg as the no-observed-adverse-effect level, which translates to an initial human dose of 5–10 mg. However, it remains uncertain whether this exposure leads to prolonged overall survival, and the optimal dose that maximizes therapeutic benefit while maintaining acceptable neurotoxicity margins has yet to be determined. Ongoing clinical trials (NCT05305885, NCT05289908, NCT03507244, NCT03101579) are currently underway to provide the necessary evidence. (Sun et al., 2010). Although isolated case reports have documented transient responses to intrathecal administration of cytarabine (Kang Xun, 2024), topotecan (Mills et al., 2024), etoposide (Park, 2015), and gemcitabine (Chen et al., 2003) in patients with NSCLC-LM, yet these anecdotes lack validation from controlled cohorts and must be interpreted with caution.

### 4.4 Intrathecal administration of alternative therapeutic agents

Recent developments have highlighted the exploration of targeted agents and immune-checkpoint inhibitors for intrathecal delivery, garnering significant attention. A Phase 1/1b trial conducted by Oliva et al. on melanoma leptomeningeal disease demonstrated the feasibility and safety of administering intrathecal nivolumab at a dose of 50 mg in conjunction with intravenous nivolumab at 240 mg every 2 weeks. Although this study focused on melanoma rather than NSCLC, the findings offer a preliminary safety framework for future biweekly administration. Although the study focused on melanoma rather than NSCLC, the findings offer a foundational safety framework for future checkpoint-inhibitor trials targeting NSCLC-LM (Glitza Oliva et al., 2023). Holdaway et al. presented a case study of a patient with glioblastoma multiforme leptomeningeal disease who underwent five cycles of intrathecal bevacizumab without experiencing severe AEs or dose-limiting toxicity. This case provides preliminary evidence of safety, suggesting the potential for further investigation of intrathecal bevacizumab in various solid tumors (Holdaway et al., 2023). Additionally, Oka K et al. detailed the case of a patient with leptomeningeal disease characterized by HER2 amplification and resistance at the primary site, who experienced rapid neurological improvement following a single 60 mg dose of intrathecal trastuzumab. This was followed by cytological clearance in the CSF at 2 months and continued intrathecal trastuzumab administration for twelve additional months without toxicity. This case substantiates the feasibility of intrathecal anti-HER2 therapy; however, its efficacy necessitates validation through further clinical research (Oka et al., 2024). These accounts, predominantly consisting of isolated case reports or small cohorts, do not confirm the safety and efficacy of intrathecal targeted agents and immune-checkpoint inhibitors. However, they collectively suggest feasibility and offer an initial framework for the development of definitive clinical trials for NSCLC-LM.

### 4.5 Intrathecal combination therapy

ITC is typically administered as monotherapy; however, research has indicated that a combination of two drugs is also feasible for patients with LM from solid tumors. Further investigation is necessary to establish the safety and efficacy of this combined approach (Scott et al., 2014). Ju et al. reported on two patients with LM from NSCLC who received a combination of intrathecal nimotuzumab and MTX. One patient was administered three weekly doses of intrathecal nimotuzumab (50 mg) in combination with MTX, and after 3 months, the administration frequency was reduced to twice weekly. This patient survived for 35 months following the diagnosis of LM and experienced no further headaches after the initiation of intrathecal therapy, with no significant intolerable adverse reactions observed. In the second patient, lower limb weakness resolved following the administration of intrathecal MTX and nimotuzumab (Jiao et al., 2016). Following three doses of intrathecal therapy, there was a notable reduction in CSF tumor markers, including carcinoembryonic antigen (CEA), which continued to decline with further treatments. Additionally, CSF pressure normalized, and cytological examination of the CSF was negative after 3 months of combined intrathecal therapy. The patient survived for 12.5 months following the diagnosis of LM. The integration of intrathecal therapy with treatments administered through alternative routes appears feasible for patients with LM originating from NSCLC. However, the efficacy of this approach and the determination of an optimal combinatorial therapeutic strategy necessitate validation through comprehensive clinical research. Huang et al. demonstrated that NSCLC-LM patients treated with ITC in conjunction with tyrosine-kinase inhibitors achieved a median overall survival of 17.0 months, significantly exceeding those who received ITC with systemic chemotherapy (7.0 months, P = 0.010) or best supportive care (6.0 months, P = 0.001). The survival advantage compared to ITC combined with PD-1 blockade was not statistically significant (Huang et al., 2024). Miao et al. conducted a prospective evaluation of pemetrexed-based intrathecal therapy, administered either sequentially or concurrently with various treatments: tyrosine-kinase inhibitors (n = 19), systemic chemotherapy (n = 10), anti-angiogenic agents (n = 10), immune checkpoint inhibitors (n = 1), whole-brain irradiation (n = 1), and bi-/tri-modality combinations (n = 13). This study reported a median progression-free survival of 9.6 months (95% CI 3.4–15.8), while overall survival data were not mature at the final follow-up. These findings highlight the significant efficacy and tolerable toxicity of intrathecal-centered multimodal therapy in patients with NSCLC-LM (Miao et al., 2020). In a related study, Zhong et al. reported a case in which a patient achieved cytological negativity following three cycles of treatment with 160 mg osimertinib and 30 mg intrathecal pemetrexed, accompanied by notable neurological improvement and an overall survival of 28 months from the diagnosis of LM (Zhong et al., 2024). Zheng et al. reported on a patient with non-small cell lung cancer with LM characterized by an EGFR-SEPT14 fusion. This patient, after undergoing multiple prior treatments, exhibited symptoms of gait ataxia, blurred vision, and progressive hearing loss. Administration of osimertinib in combination with intrathecal pemetrexed at a dosage of 50 mg on days 1 and 8 of a 21-day cycle successfully alleviated these symptoms, resulted in negative CSF cytology, and did so without significant toxicity. Eight months post-treatment, the patient maintained complete intracranial and extracranial remission according to RANO criteria (Zheng et al., 2021). Additionally, a 52-year-old patient whose initial presentation of EGFR L858R-mutant NSCLC was a severe meningeal syndrome—characterized by a CSF opening pressure exceeding 250 mmH_2_O, malignant cytology, and a carcinoembryonic antigen (CEA) level of 707.8 ng/mL—achieved rapid symptom relief with high-dose almonertinib at 165 mg in conjunction with pemetrexed delivered via an Ommaya reservoir at 30 mg on days 1 and 8. Within 1 week, symptoms of intracranial hypertension, headache, and emesis subsided, and by the second week, the CSF CEA level had decreased to 138.5 ng/mL. This outcome supports the efficacy of intensified EGFR blockade combined with reservoir-based ITC as a viable rescue strategy for NSCLC-LM presenting with severe meningeal crisis (Wang W. et al., 2023). Current prospective trials, including NCT05305885 and NCT03507244, are investigating the potential of intrathecal pemetrexed combined with cranial irradiation to extend survival in patients with LM. However, regardless of whether the approach involves the combination of two intrathecal agents or integrates intrathecal administration with systemic or radiation therapies, there is a lack of large-scale controlled data. As a result, no standard-of-care protocol has been established, and clinical practice must await more robust evidence.

### 4.6 Adverse reactions of intrathecal administration

The efficacy of ITC in patients with LM is well-established; however, the safety of these treatments remains a paramount concern. Prior to the administration of ITC, a thorough assessment of the CSF pathways is imperative. In cases where CSF flow is abnormal, intrathecal administration can result in elevated drug concentrations at the site of obstruction, potentially leading to unexplained toxicity (Kumar et al., 2021). Intrathecal injections are typically delivered via two methods: the Ommaya reservoir or lumbar puncture (Ozcan et al., 2023). Lumbar puncture, a common clinical procedure, involves the insertion of a needle into the intervertebral space of the lumbar spine to access the subarachnoid space, followed by the gradual administration of chemotherapeutic agents. Although this method is relatively straightforward, the requirement for repeated punctures may increase patient discomfort and the risk of infection. Furthermore, the amount of medication that can be administered in a single session is limited, potentially necessitating multiple injections to achieve the desired therapeutic outcome. The Ommaya reservoir is a medical device designed to facilitate access to the intracranial CSF space. One end of the reservoir is connected to this space, while the other end is subcutaneously tunneled to extend outside the body. During implantation, a small aperture is created in the patient’s skull to accommodate the reservoir within the cranium. This setup allows for the direct administration of chemotherapeutic agents into the CSF, thereby specifically targeting neoplastic cells located within the meninges and CSF. The use of the Ommaya reservoir mitigates the discomfort and infection risks associated with repeated lumbar punctures and enables precise regulation of drug dosage and administration timing. Empirical studies suggest that patients receiving intraventricular chemotherapy via the Ommaya reservoir experience significant improvements in both therapeutic outcomes and quality of life. Nonetheless, the surgical implantation of the Ommaya reservoir is not without risks, as it may result in complications such as hemorrhage and infection. Additionally, post-implantation challenges may include reservoir obstruction and displacement (Ozcan et al., 2023). The personalized selection of drug administration methods is clinically significant in reducing adverse reactions in patients. The side effects associated with ITC can be broadly classified into three categories: acute, subacute, and chronic toxic reactions. Acute toxic reactions typically present as transient neurological impairments, occurring 2–4 h post-administration, and are characterized by symptoms such as dizziness, headache, nausea, back pain, and fever, usually persisting for approximately 12–72 h (Wu Xi and Xiao, 2019; Hou et al., 2021). Subacute toxic reactions typically manifest within a timeframe of days to weeks following intrathecal administration, and are characterized by cranial nerve palsies, seizures, weakness, or ataxia. In contrast, chronic toxic reactions, predominantly leukoencephalopathy, emerge months to years post-injection. The primary clinical adverse effects associated with intrathecal MTX administration include myelosuppression, gastrointestinal disturbances, leukoencephalopathy, seizures, abnormalities in respiratory rhythm, numbness in the lower limbs, and mild pain. Most patients generally tolerate these adverse effects, and the majority of these reactions are reversible (Hou et al., 2021; Wang et al., 2017). Subacute toxic reactions emerge within days to weeks following ITC and may include cranial nerve palsies, seizures, weakness, or ataxia. Chronic toxic reactions, such as leukoencephalopathy, manifest months to years after intrathecal injections. Myelosuppression is the most prevalent toxicity, with an incidence ranging from 10.34% to 78.4%. Notably, in the retrospective study conducted by Huang et al., the incidence of myelosuppression reached 78.4% with the MTX plus tyrosine kinase inhibitor regimen, marking the highest rate among all intrathecal protocols and primarily manifesting as leukopenia and thrombocytopenia. Leukoencephalopathy is the characteristic chronic neurotoxicity, with an incidence ranging from 23% to 68%. In two phase II single-arm studies conducted by Pan et al., the incidence of leukoencephalopathy with MTX plus radiotherapy regimens was reported at 23% and 68%, respectively. This condition presents as cognitive decline, ataxia, seizures, and other neurological deficits, which are associated with drug accumulation in brain tissue and subsequent damage to the myelin sheath. Additional toxicities include gastrointestinal disorders (incidence of 40.5%), radiculitis (13%–27%), and mucositis (11%–20%), most of which are characterized as mild to moderate acute toxicities (Wu Xi and Xiao, 2019; Scott et al., 2014; Wang et al., 2017; Pan et al., 2023). The adverse reactions predominantly associated with intrathecal administration of pemetrexed encompass myelosuppression, neurotoxicity, leukoencephalopathy, elevated transaminases, weight loss, headaches, nausea and vomiting, and seizures. Myelosuppression is the main toxicity, occurring in 26.08%–36.7% of cases, mainly as anemia, thrombocytopenia, and leukopenia. Hong et al. reported anemia and thrombocytopenia incidences of 61.4% and 40.4%, mostly Grade 1–2, with severe cases being rare. Neurotoxicity, including limb paresthesia (10.5%), headache (10.0%), and seizures, is linked to effects on neuronal metabolism in the CSF and is less common than with MTX. Elevated transaminases (26.1%) are the main hepatotoxicity, typically transient, with no severe liver injury reported. Other mild toxicities, such as nausea and vomiting (20.0%) and limb pain (16.7%), resolve quickly after stopping the drug. Most of these AEs are mild and reversible, with the majority resolving following symptomatic treatment (Fan et al., 2021; Zy Pan and Jiang, 2022; Fan et al., 2024).

Research by Holdaway et al. demonstrates that no adverse reactions were observed at any dosage level when bevacizumab was administered intrathecally, starting with a 25 mg dose and increasing by 12.5 mg every 2 weeks to a maximum of 50 mg. These findings suggest that intrathecal bevacizumab is safe (Holdaway et al., 2023). Additionally, Oliva et al.'s study reports that with the intravenous administration of nivolumab at a dosage of 240 mg, combined with a biweekly intrathecal injection of nivolumab (50 mg), the most common grade 1 or 2 AEs include nausea (36%), diarrhea (24%), lymphopenia (24%), elevated aspartate aminotransferase and/or alanine aminotransferase (ALT) levels (24%), and papular rash (24%) (Glitza Oliva et al., 2023).

## 5 Limitations

While this study systematically reviewed the clinical evidence regarding the use of ITC for treating LM in non-small cell lung cancer, thereby providing a valuable reference for clinical practice, it possesses several inherent limitations that necessitate further refinement in future research. Firstly, the design types of the included studies exhibited significant bias; among the 12 studies, single-arm phase I/II trials and retrospective cohort studies were predominant, with a notable absence of high-quality randomized controlled trials. Additionally, some studies had small sample sizes, with a minimum of only 19 cases, complicating the exclusion of selection bias and information bias, which in turn diminishes the strength of the evidence and its generalizability. Secondly, there was considerable heterogeneity across the studies: treatment regimens varied widely, including monotherapy, combination therapy, intrathecal combined with systemic therapy or radiotherapy, and other modalities, with significant differences in drug dosage, administration cycles, and infusion routes. Stratified analyses based on prior treatment lines and performance status were not conducted, thereby hindering the precise evaluation of therapeutic benefits across different patient subgroups. Furthermore, the reporting of efficacy and safety endpoints was incomplete and inconsistent. Specifically, only three studies reported progression-free survival, and some studies did not provide detailed assessment criteria for objective response rate and disease control rate. Additionally, AEs recording was largely restricted to incidents with an incidence rate of ≥10%, lacking a systematic description of AEs severity, onset timing, interventions, and outcomes. Moreover, there was no consideration of patient quality of life or neurological function improvement as clinical endpoints, complicating the comprehensive evaluation of the clinical value of ITC.

## 6 Summary

LM represents a particularly challenging complication of NSCLC, yet recent clinical investigations into intrathecal injection have demonstrated certain therapeutic advantages. To enhance the survival prognosis of patients with NSCLC-associated LM, a growing body of research is exploring the use of various chemotherapeutic agents, targeted therapies, and immune checkpoint inhibitors (ICIs) for intrathecal administration. Additionally, there is an increasing number of clinical studies examining the efficacy of combining intrathecal injection with other therapeutic modalities. Analysis of ITC regimens shows MTX (10–15 mg) and pemetrexed (10–50 mg) are common, while immune checkpoint inhibitors are given at fixed exploratory doses. High-dose combinations with targeted therapy or radiotherapy are more effective, and the Ommaya reservoir is ideal for long-term treatment. Insufficient dosing leads to poor outcomes, while excessive dosing increases toxicity and risks. Optimal doses for each agent are still undefined, requiring personalized adjustments based on patient factors. Clinical trials are investigating dose optimization, but current protocols lack standardization. Large-scale randomized controlled trials are needed. Despite these efforts, the majority of current research on intrathecal injection therapy for NSCLC-LM is retrospective in nature, with a notable scarcity of prospective studies and generally small sample sizes. Consequently, the effectiveness of intrathecal therapy for LM in NSCLC, along with the efficacy and safety of the optimal intrathecal injection regimen, necessitates further validation through additional clinical trials. Simultaneously, careful consideration is advised when translating the findings of this study into clinical practice.
